# Classification of internet addiction using machine learning on electroencephalography synchronization and functional connectivity

**DOI:** 10.1017/S0033291725001035

**Published:** 2025-05-16

**Authors:** Hsu-Wen Huang, Po-Yu Li, Meng-Cin Chen, You-Xun Chang, Chih-Ling Liu, Po-Wei Chen, Qiduo Lin, Chemin Lin, Chih-Mao Huang, Shun-Chi Wu

**Affiliations:** 1 National Center for Geriatrics and Welfare Research, National Health Research Institutes, Zhunan, Taiwan; 2 Center for Intelligent Drug Systems and Smart Bio-devices (IDS^2B^), National Yang Ming Chiao Tung University, Taiwan; 3 Department of Eengineering and System Science, National Tsing Hua University, Hsinchu, Taiwan; 4 Department of Linguistics and Translation, City University of Hong Kong, Hong Kong; 5 Department of Psychiatry, Keelung Chang Gung Memorial Hospital, Keelung City, Taiwan; 6 College of Medicine, Chang Gung University, Taoyuan County, Taiwan; 7 Department of Biological Science and Technology, National Yang Ming Chiao Tung University, Hsinchu, Taiwan

**Keywords:** Internet addiction, *k*-nearest neighbor classification, machine learning, phase lag index, random forest, support vector machine, weighted-phase lag index

## Abstract

**Background:**

Internet addiction (IA) refers to excessive internet use that causes cognitive impairment or distress. Understanding the neurophysiological mechanisms underpinning IA is crucial for enabling an accurate diagnosis and informing treatment and prevention strategies. Despite the recent increase in studies examining the neurophysiological traits of IA, their findings often vary. To enhance the accuracy of identifying key neurophysiological characteristics of IA, this study used the phase lag index (PLI) and weighted PLI (WPLI) methods, which minimize volume conduction effects, to analyze the resting-state electroencephalography (EEG) functional connectivity. We further evaluated the reliability of the identified features for IA classification using various machine learning methods.

**Methods:**

Ninety-two participants (42 with IA and 50 healthy controls (HCs)) were included. PLI and WPLI values for each participant were computed, and values exhibiting significant differences between the two groups were selected as features for the subsequent classification task.

**Results:**

Support vector machine (SVM) achieved an 83% accuracy rate using PLI features and an improved 86% accuracy rate using WPLI features. *t*-test results showed analogous topographical patterns for both the WPLI and PLI. Numerous connections were identified within the delta and gamma frequency bands that exhibited significant differences between the two groups, with the IA group manifesting an elevated level of phase synchronization.

**Conclusions:**

Functional connectivity analysis and machine learning algorithms can jointly distinguish participants with IA from HCs based on EEG data. PLI and WPLI have substantial potential as biomarkers for identifying the neurophysiological traits of IA.

## Introduction

In recent years, the field of computer science has undergone considerable expansion, leading to a rapid increase in Internet utilization, particularly among teenagers and college students(Chou, Condron, & Belland, 2005; Widyanto & Griffiths, 2006). Despite the numerous benefits it has brought to our lives, excessive Internet use is considered an emerging psychiatric disorder (Meng et al., 2022; Olson et al., 2022). Internet addiction (IA), which refers to extended and excessive Internet usage, can give rise to addictive tendencies toward Internet use, exerting a considerable impact on various aspects of young adults’ lives, such as their interpersonal relationships, academic performance, and overall physical and mental well-being. Although there is still debate regarding whether IA should be included as a distinct disorder in the Diagnostic and Statistical Manual of Mental Disorders Fifth Edition (American Psychiatric Association, 2013), IA clearly appears to be a growing global issue. Pending further research and evidence, IA remains a significant problem.

Common signs of IA include prolonged online engagement, an inability to curb the urge to go online despite recognizing its negative consequences, and discomfort when disconnected from the Internet (Chou, Condron, & Belland, 2005; Widyanto & Griffiths, 2006). Studies have also linked IA to co-occurring conditions such as attention deficit hyperactivity disorder (Ko et al., 2008; Yoo et al., 2004), depression (Ha et al., 2007; Kim et al., 2006; Morrison & Gore, 2010), anxiety (Bernardi & Pallanti, 2009), and obsessive-compulsive disorder (Zhang, Amos, & McDowell, 2008). Some researchers view IA as akin to an addictive behavior (Hall & Parsons, 2001; Holden, 2001), sharing clinical parallels with pathological gambling (Lee et al., 2012) and substance use disorders (Sharifat, Rashid, & Suppiah, 2018). These parallels involve challenges in impulse control (Grant et al., 2010) and a lack of self-control regarding substance, alcohol, or Internet usage. Although many recent studies using frequency power analysis have examined the neurophysiological traits of IA, their findings often vary, with biomarkers appearing in different bands. Furthermore, this approach does not account for interactions between brain regions, highlighting the need for further research to identify reliable biomarkers, particularly those examining brain region interactions, to enhance clinical diagnosis and support early treatment of IA (Park et al., 2018).

The diagnosis of IA often relies on self-assessment questionnaires, such as Young’s IA Test (Young, 1996) or the Chen IA Scale (CIAS) (Chen et al., 2003). While these questionnaires provide valuable subjective insights, resting-state electroencephalography (EEG) offers an objective approach to measuring brain activity, enabling a deeper understanding of the neural mechanisms underlying IA. Resting-state EEG, a noninvasive method, measures the collective electrical potential generated by neuronal activity in a relaxed, awake state. Given that EEG comprises various frequencies, each linked to specific cognitive functions (Patil et al., 2022), it is increasingly recognized as a powerful tool for studying the neural correlates of cognition and behavior. Furthermore, EEG provides neurophysiological markers that may not be captured by self-report measures alone (Patil et al., 2022).

Research has linked specific EEG frequency bands to various cognitive and emotional states: delta activity (1–4 Hz) is associated with sensory afferent inhibition (Harmony, 2013); theta activity (4–8 Hz) with nervousness (Wang et al., 2015); alpha activity (8–12 Hz) with relaxation (Klimesch, 1999); beta activity (12–30 Hz) with attention (Huster et al., 2013); and gamma activity (30–60 Hz) with inhibitory control (Modolo et al., 2020). The utility of resting EEG has been demonstrated in diagnosing and studying clinical conditions such as epilepsy (Huster et al., 2013), brain tumors (Liu et al., 2020), and sleep disorders (Peter-Derex et al., 2021). Resting-state EEG has been employed to identify IA (Choi et al., 2013; Kim et al., 2017; Lee et al., 2014). For instance, Choi et al. (2013) utilized absolute and relative power analysis to study individuals with IA, revealing reduced absolute beta power and increased absolute gamma power across the scalp. These EEG band activities were significantly correlated with IA severity and impulse control. However, other studies have reported variations in delta and theta power among IA participants (Kim et al., 2017; Lee et al., 2014). Such discrepancies in frequency power analysis results may arise from differences in EEG features and algorithms used across studies. While power analysis reveals neural oscillation activity in different frequency bands, it does not capture the direction of signal phases or interregional connections, which could provide critical cognitive insights related to addiction symptoms.

EEG functional connectivity examines interactions between neurons across brain regions, providing insights into the neural networks underlying IA. Research has identified deficits in functional connectivity in individuals with IA, particularly in brain networks critical for cognitive functioning (Park et al., 2018). A study on individuals with Internet gaming disorder (IGD) has reported increased connectivity within the default mode network (DMN; theta, alpha, and beta bands) and the reward-salience network (RSN; alpha and beta bands) (Lee et al., 2022), highlighting the involvement of these networks in impaired cognitive and reward processing associated with IGD. Additionally, altered connectivity patterns have been linked to gaming behaviors, suggesting potential neurophysiological markers for IGD. The relationship between IA and brain network topology during working memory tasks has been explored using EEG and graph theory analysis (Wang et al., 2020). Individuals with IA exhibited higher global efficiency and network hierarchicality. They also showed stronger functional connectivity integration, particularly in prefrontal and limbic regions, which may support enhanced working memory performance. Other studies have reported increased intrahemispheric coherence in the beta and gamma bands among IA participants (Park et al., 2017, 2018), implicating the brain’s reward system, cognitive functions, and impulse control mechanisms (Ding et al., 2014). Synchronization measures, however, are susceptible to volume conduction and reference effects, leading to the adoption of metrics like the phase lag index (PLI) for more reliable connectivity estimates (Stam, Nolte, & Daffertshofer, 2007). For instance, a study using PLI to investigate intrabrain connectivity during a ‘Stop’ signal task revealed significantly greater connectivity in multiple brain regions among individuals with IA compared to healthy controls (HCs) (Su et al., 2023). These findings highlight notable differences in brain interconnections, further emphasizing the importance of altered connectivity patterns in IA.

Recently, machine learning has become increasingly prominent in IA research, supporting the classification and prediction of IA using EEG. For example, Gross, Baumgartl, and Buettner (2020) employed random forest algorithms to identify frequency bands significantly associated with IA, using power values as features to classify individuals as IA or non-IA. Similarly, Wang et al. (2021) applied support vector regression to analyze changes in functional connectivity, predicting behavioral score variations and assessing the effectiveness of cognitive behavioral therapy for IA. Deep learning methods, such as convolutional neural networks, have also been utilized to distinguish individuals with IA from controls, leveraging EEG for advanced pattern recognition (Sun et al., 2022). These studies highlight the potential of integrating EEG markers with machine learning to enhance IA diagnosis and treatment evaluation.

The current study aimed to implement the PLI and weighted PLI (WPLI) to examine functional connectivity in individuals with IA and HCs. Phase synchronization has been proposed as a vital mechanism for establishing communication networks among different brain regions (Engel, Fries, & Singer, 2001; Fries, 2005). The PLI and WPLI serve to quantify the extent of phase synchronization among various brain regions, rendering them unaffected by inter-individual variations in power intensity. Compared with alternative metrics of phase synchronization, such as the phase locking value (Lachaux et al., 1999) or the imaginary component of coherence (Nolte et al., 2004), the PLI and WPLI display heightened resilience against the impact of volume conduction (Stam, Nolte, & Daffertshofer, 2007). This phenomenon arises when a dominant source in the brain inaccurately triggers phase synchrony. Through the utilization of the PLI and WPLI, our objective was to pinpoint the key characteristics of IA and evaluate the reliability of these attributes as potential biomarkers for classification across various machine-learning methods.

## Methods

### Participants

This study recruited 96 participants, consisting of 47 males and 49 females, all within the age range of 18 to 25. All participants provided written informed consent prior to their participation, and the study was approved by the Human Subject Ethics Committee of the City University of Hong Kong. The authors assert that all procedures contributing to this work comply with the ethical standards of the relevant national and institutional committees on human experimentation and with the Helsinki Declaration of 1975, as revised in 2008. IA severity was assessed using the CIAS (Chen et al., 2003), which consists of 26 questions. The participants assessed each question’s alignment with their personal circumstances, with responses rated on a scale from 1 to 4, where 4 indicated the highest degree of concordance. These questions encompassed diverse facets such as Internet usage patterns and the influence of the Internet on daily life and its repercussions on health. The CIAS has demonstrated excellent reliability (Cronbach’s α = 0.94) and strong correlations with IA-related behaviors, such as time spent online, supporting its clinical and research applications (Ko et al., 2005, 2005). Cronbach’s α (ranging from 0 to 1) measures internal consistency, with values ≥0.9 indicating high reliability.

Following the criteria outlined by Ko et al. (2005), participants were divided into two groups: the IA group, comprising individuals with a CIAS score of 64 or higher, and the HC group, with scores below 64. This threshold was selected based on findings demonstrating a robust Cohen’s Kappa value of 0.61, indicating substantial agreement between the CIAS score and clinical diagnoses and underscoring its reliability as a diagnostic tool. Additionally, a score of 64 achieved the highest diagnostic accuracy (87.6%) and strong specificity (92.6%). The diagnostic odds ratio (DOR) of 26.17 further highlights the strong discriminatory power of this threshold, reinforcing its value in both research and clinical applications. Finally, the CIAS threshold was validated in college students (Ko et al., 2009), further supporting its relevance to this study. After the exclusion of four subjects due to data quality issues, the study ultimately included 42 subjects (20 females) in the IA group (CIAS mean score 75.9) and 50 subjects (27 females) in the HC group (CIAS mean score 54.1). Importantly, no significant differences were found between the two groups in gender, age, or handedness. Statistical analyses showed comparable gender distribution (HC: 23 males/27 females; IA: 21 males/21 females, *χ^2^* = 0.03*, p* = 0.863), handedness distribution (HC: 47 right-handed/3 left-handed; IA: 41 right-handed/1 left-handed, *χ^2^* = 0.112*, p* = 0.738), and age (HC: 20.56 ± 1.57; IA: 20.71 ± 1.50, *t* = 0.48, *p* = 0.633) across groups. Notably, all participants were well-controlled university students from the same population, ensuring consistency in sample characteristics and minimizing external variability. A summary of these comparisons is provided in Table 1.

**Table 1.** Demographic and clinical characteristics of the HC and IA groups

	HC	IA	Statistics	*p*-value
	N = 50	N = 42		
Age	20.56 ± 1.57	20.71 ± 1.50	t = 0.48	0.633
Gender (male/female)	23/27	21/21	χ^2^ = 0.03	0.863
Handedness (right/left)	47/3	41/1	χ^2^ = 0.112	0.738
CIAS	53.52 ± 7.14	76.14 ± 7.35	t = 14.94	0.000***

*** *p* < .001

### Data acquisition

Resting-state EEG was recorded for 5 minutes (with eyes open) using a cap with 32 Ag/AgCl electrodes (QuikCap, Compumedics Neuroscan). The signals were amplified using a NuAmps amplifier (Compumedics Neuroscan) with a band-pass filter of 0.1–100 Hz and digitized at a rate of 1000 Hz. The data were referenced to the average signals of the left and right mastoids. Horizontal eye movement artifacts were tracked using two electrodes positioned at the outer canthi of both eyes, while vertical eye movement was monitored using two electrodes placed above and below the left eye. The impedance of all electrodes was carefully maintained below 5 kΩ to ensure the acquisition of high-quality data. The participants were explicitly instructed to remain awake and relaxed throughout the recording session.

### EEG preprocessing

The raw data underwent several processing steps to enhance its quality. Initially, a finite impulse response (FIR) band-pass filter in the range of 1 to 60 Hz was applied to exclude noise outside the target frequency range. Subsequently, a 49–51-Hz FIR band-stop filter was used to mitigate the influence of power line interference. Thereafter, a manual examination was conducted to detect any segments of data contaminated by significant artifacts, including body movements, eye blinks, and environmental factors. These contaminated data segments were then systematically removed from the time series. Participants with data segments shorter than 10 seconds were excluded during this stage. To further refine the data, we employed independent component analysis (ICA) (Bell & Sejnowski, 1995) as a blind source separation technique to distinguish brain signals from various artifacts. ICA proved particularly effective in eliminating artifacts that could not be directly addressed in the prior steps, such as long-lasting muscle or heart signals. We used the IC label (Delorme & Makeig, 2004) to identify artifact components and then exclude them from the dataset. All of these preprocessing procedures were executed using MATLAB R2020a (MathWorks) and EEGLABv2020.1 (Delorme & Makeig, 2004) to ensure the integrity and quality of the data.

### Phase lag index

To calculate the PLI, first, the analytical signal 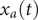 must be constructed from the preprocessed EEG data series 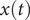 (Bruns, 2004):
(1)

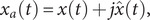

where 
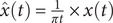
 is the Hilbert transform of 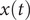 , and the symbol 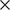 denotes the convolution operator. Equation (1) shows that the analytical signal comprises both the real component of the original signal and its corresponding Hilbert transform within the imaginary component. The instantaneous phase 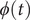 of 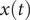 can then be determined using the following equation:
(2)

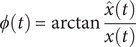

Using Equation (2), the PLI between any two EEG channels can be computed as follows:
(3)

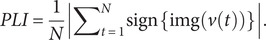

**Figure 1.**
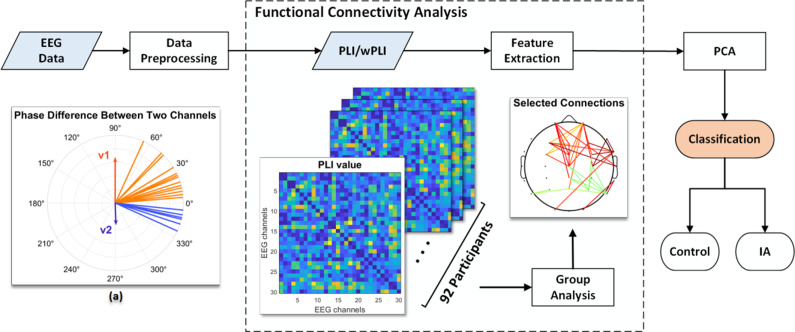
The entire procedure of data preprocessing and classification. (a) The distribution of an example *v*(*t*) suggesting the presence of a phase delay at certain degrees. In this example, the uneven length of *v*1 and *v*2 indicates the asymmetry in phase differences, and the PLI value is 0.3.

Here, 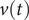 is defined as 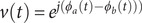 , where 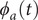 and 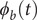 represent the instantaneous phases of channels *a* and *b*, respectively. Further notations are as follows: 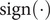 signifies the sign function, 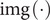 indicates the projection of 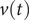 onto the imaginary axis, and *N* corresponds to the length of the time series. Figure 1 illustrates the distribution of an example 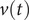 . Notably, we can observe a higher concentration of 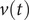 in the range of -*π* to 0 degrees than in the range of 0 to *π* degrees, indicating the presence of a phase delay at specific degrees. The PLI value falls within the range of 0 to 1. PLI values are elevated when the instantaneous phase differences between two channels consistently exhibit a persistent alignment in the same direction over time, indicating a substantial phase lead or delay.

### Weighted phase lag index (WPLI)

The WPLI (Vinck et al., 2011) extends the PLI by considering the magnitude of the imaginary component of 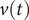 . It assigns weights to the contributions of observed phase leads and lags and can be calculated using the following formula:
(4)

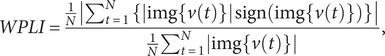

where the denominator in the equation functions to normalize the magnitude of the weighted imaginary component in the numerator. This normalization process ensures that the resulting value falls within the range of 0 to 1.

PLI is prone to noise-induced fluctuations, particularly when phase differences approach 0 or π. It is also vulnerable to volume conduction, which can produce spurious correlations from shared sources rather than genuine neural connectivity (Vinck et al., 2011). To address these challenges, WPLI incorporates an amplitude-weighting mechanism that reduces noise sensitivity and minimizes volume conduction effects by assigning lower weights to phase interactions with minimal imaginary components. Comparative studies have validated WPLI’s advantages over PLI (Yoshinaga et al., 2020; Yu, 2020). They benchmarked functional connectivity metrics in simulated and real EEG/MEG data, demonstrating WPLI’s superiority in handling noise, particularly at lower levels, and achieving higher test–retest reliability. Hardmeier et al. (2014) further showed that WPLI more effectively differentiates frequency-specific connectivity patterns across multiple bands and reduces false-positive connectivity from near-zero phase lags. These findings highlight WPLI’s robustness, sensitivity to synchronization transitions, and reliability in real-world conditions.

### Machine learning methods

1. Support vector machine (SVM)

The SVM (Cortes & Vapnik, 1995) is a supervised learning algorithm renowned for its effectiveness in tackling complex classification problems. Its primary objective is to discern a hyperplane within the 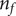 -dimensional feature space (where 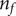 represents the number of features) that adeptly separates distinct classes. This hyperplane functions as the decisive boundary, with the closest samples to it being termed support vectors. Notably, the SVM leverages the kernel method, allowing it to project data into a higher-dimensional feature space. This capability empowers the SVM to identify an even more optimal hyperplane for the effective separation of data from diverse classes. In our study, we employed the widely utilized radial basis function (Orr, 1996) as the kernel function.

1. Random Forest (RF)

RF is another potent supervised learning algorithm that employs the concept of bootstrap aggregation (Breiman, 1996), commonly referred to as an ensemble learning technique. This method entails the independent training of multiple decision tree models. In the case of each decision tree, the original dataset undergoes random sampling to form subsets for training. When performing classification tasks, the ultimate outcome is determined by amalgamating the individual decisions made by each decision tree via a voting mechanism. This ensemble approach equips the RF with the ability to adeptly handle noisy and high-dimensional data with intricate interrelationships. Furthermore, it showcases reduced vulnerability to overfitting when compared with the utilization of a single decision tree.

1. *k*-Nearest Neighbor (*k*NN)

The *k*NN (Altman, 1992) represents a straightforward supervised learning algorithm that stands apart from the intricacies of the previously discussed methods. In classification tasks, the class of a new sample is established by assessing the predominant class among its *k* closest neighbors, with *k* representing the number of neighboring samples considered. Diverse techniques exist for quantifying the distance between samples in the *k*NN. In this study, we employed the Euclidean distance, a widely used and straightforward method. This distance metric computes the straight-line distance between two samples within the feature space.

### Implementation details

We computed PLI and WPLI values for each participant, encompassing all channel pairs across eight frequency bands: delta (1–4 Hz), theta (4–8 Hz), alpha (8–12 Hz), beta1 (12–21 Hz), beta2 (21–30 Hz), gamma1 (30–40 Hz), gamma2 (40–50 Hz), and gamma3 (50–60 Hz). Next, we employed the two-sample *t*-test to identify connections characterized by the participants’ mean PLI or WPLI values that exhibited a statistically significant difference between the IA and HC groups, with a significance threshold set at *p* < 0.05. The PLI and WPLI values exhibiting statistical significance were selected as features for the subsequent classification task. To standardize the scale of each feature, we utilized the z-score transformation (Cheadle et al., 2003), which centers each feature around a mean of zero and scales it to have a standard deviation of 1. We then applied principal component analysis (PCA) (Jolliffe & Cadima, 2016) for dimensionality reduction. This step aimed to retain informative components in the selected features while eliminating noise and redundant information. By implementing PCA, we simultaneously reduced the training time and enhanced the performance. The decision regarding the number of principal components to retain in the PCA was determined based on the cumulative explained variance ratio, which was set at 99%.

We conducted model training and evaluation using a five-fold cross-validation approach (Kohavi, 1995). The dataset was randomly divided into five subsets, with efforts made to maintain a roughly equal class distribution in each subset. During each trial, four of these subsets were employed as training sets, while one subset served as the testing set. This process was repeated five times, with each subset taking on the role of the testing set once. Subsequently, the outcomes from the five testing sets were averaged to derive the final result. For the classification task, we employed three machine learning algorithms, as discussed previously. All methodologies were implemented using Python 3.6 and the Keras 2.1 API, ensuring a robust and standardized environment for experimentation and analysis. The entire procedure is illustrated in Figure 1.

## Results

### Statistically significant connections

**Figure 2.**
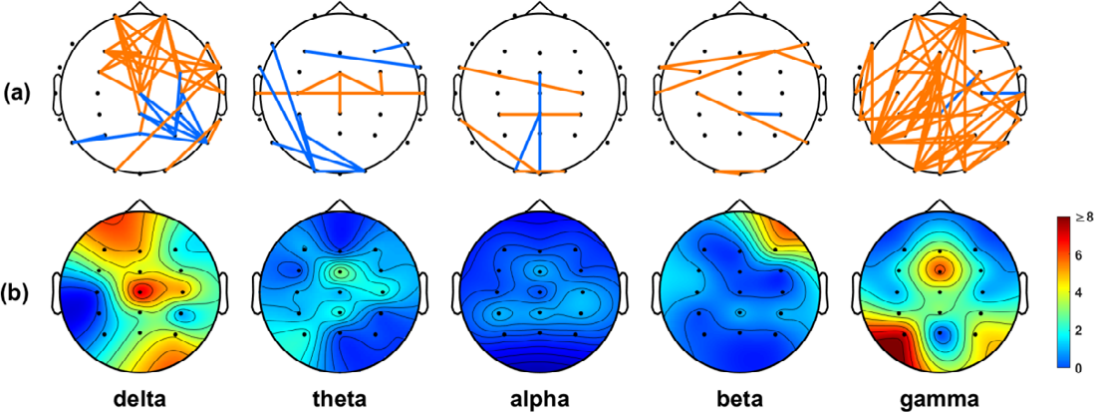
(a) Significant connections identified based on the PLI values between the IA and HC groups. Orange lines indicate the connections that were stronger in the IA group than in the HC group, while blue lines indicate the connections that were weaker in the IA group than in the HC group. (b) Electrode engagement map showing the number of significant connections calculated for each electrode.

We employed the *t*-test to identify connection differences between the IA and HC groups, using a lenient significance threshold (*p* < 0.05) without adjusting for multiple comparisons. This approach allowed us to include most of the connections based on their PLI values. Figure 2a shows that the IA group had stronger connections than the HC group in numerous regions (illustrated in orange). Notably, a significant proportion of these connections were consistently observed in both the delta and gamma frequency bands. In the delta band, we detected numerous connections originating from electrodes placed on the frontal region (Figure 2b). The gamma band analysis revealed a significant number of connections originating from electrodes distributed across the entire scalp, with many of them placed on the occipital area (Figure 2b).

**Figure 3.**
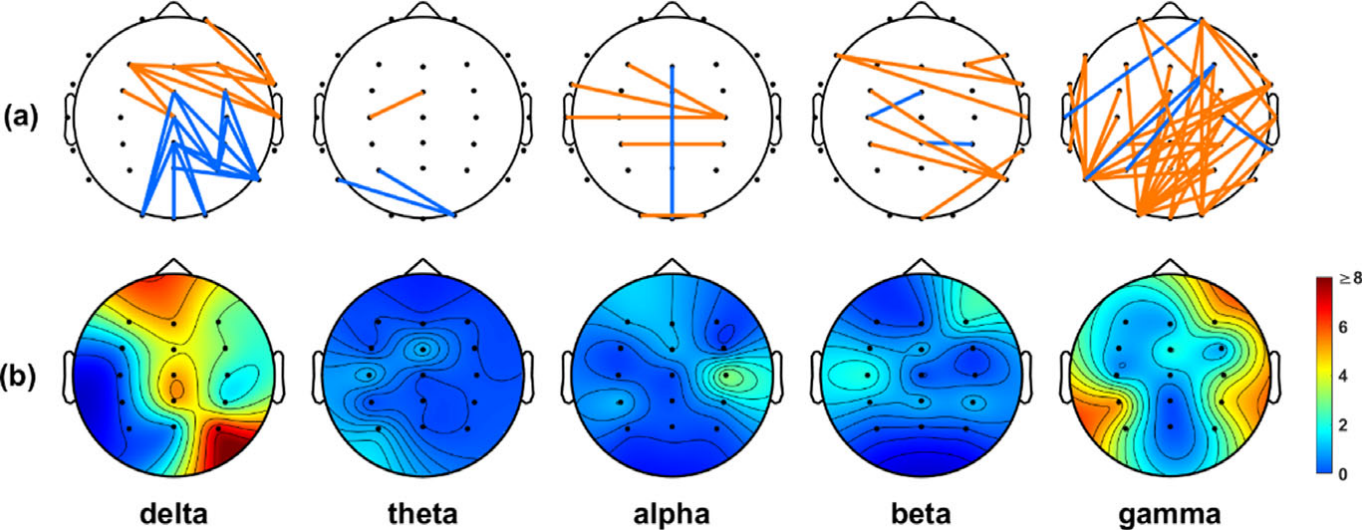
(a) Significant connections identified based on the WPLI values between the IA and HC groups. Orange lines indicate the connections that were stronger in the IA group than in the HC group, while blue lines indicate the connections that were weaker in the IA group than in the HC group. (b) Electrode engagement map showing the number of significant connections calculated for each electrode.

In the context of the WPLI analysis, our findings in Figure 3 closely echoed the patterns depicted in Figure 2, emphasizing a preponderance of significant connections within the delta and gamma frequency bands. Furthermore, both the PLI and WPLI showed elevated values in the IA group, indicating the potential presence of increased neuronal synchronization within this group.

### Classification results

We used three performance metrics, namely accuracy, sensitivity, and specificity, to evaluate the performance of the three classification models (SVM, RF, and *k*NN). Definitions for each of these metrics are presented below.
(5)

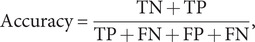



(6)

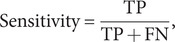



(7)

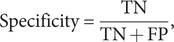

where TN represents ‘True Negative,’ TP represents ‘True Positive,’ FN represents ‘False Negative,’ and FP represents ‘False Positive.’ ‘True Positive’ is defined as the accurate classification of an IA subject into the IA group. Accuracy measures the probability of accurate predictions across all samples; sensitivity quantifies the probability of accurate predictions within the IA group; and specificity quantifies the probability of accurate predictions within the HC group.

Table 2 presents the means and standard deviations of the evaluation results obtained from five datasets using PLI values as features. Among the three models, the SVM model demonstrated superior performance, achieving an accuracy rate of 83%. The SVM model also had higher sensitivity values than the *k*NN and RF models, suggesting that the SVM model was more efficient than the other models in identifying the IA group. Moreover, the SVM model showed similar performance to the other models in classifying the IA (sensitivity measure) and HC (specificity measure) groups. In contrast, the *k*NN and RF models showed better performance in classifying the HC group than in classifying the IA group.

**Table 2.** The results of classification performed using PLI values as the feature set

	SVM	*k*NN	RF
Accuracy	0.83 ± 0.12	0.78 ± 0.15	0.79 ± 0.16
Sensitivity	0.80 ± 0.11	0.71 ± 0.1	0.67 ± 0.12
Specificity	0.83 ± 0.07	0.84 ± 0.08	0.90 ± 0.08
TN	8.6 ± 1.04	8.40 ± 1.02	9.00 ± 1.2
FP	1.40 ± 1.04	1.60 ± 1.02	1.00 ± 1.2
FN	1.80 ± 1.3	2.40 ± 1.25	2.80 ± 1.4
TP	6.60 ± 1.14	6.00 ± 1.25	5.60 ± 1.33

Table 3 presents the outcomes obtained when utilizing WPLI values as the feature set. Upon integrating the WPLI into the SVM model, we observed an improvement in performance, resulting in an average accuracy of 86%. Nevertheless, we continued to observe lower sensitivity values for both the *k*NN and RF models than for the SVM model, as previously noted. This means that the SVM model performed better than the *k*NN and RF models in classifying the IA group. One possible factor contributing to this sensitivity difference is the use of the kernel function in the SVM model. This kernel function transforms features into a higher-dimensional space, potentially enhancing the model’s capacity to distinguish between the two data groups.

**Table 3.** The results of classification performed using WPLI values as the feature set

	SVM	*k*NN	RF
Accuracy	0.86 ± 0.05	0.81 ± 0.11	0.80 ± 0.06
Sensitivity	0.88 ± 0.11	0.80 ± 0.17	0.71 ± 0.16
Specificity	0.84 ± 0.05	0.82 ± 0.10	0.88 ± 0.10
TN	8.40 ± 0.49	8.20 ± 0.98	8.80 ± 0.98
FP	1.60 ± 0.49	1.80 ± 0.98	1.20 ± 0.98
FN	1.00 ± 0.89	1.60 ± 1.36	2.40 ± 1.20
TP	7.40 ± 1.36	6.80 ± 1.72	6.00 ± 1.67

## Discussion

In this study, we employed the PLI and WPLI to investigate phase synchronization in the EEG patterns of individuals with and without IA. Notably, we observed analogous topographical patterns in the results of the *t*-test for both the WPLI and PLI. Of special significance was the identification of numerous connections within the delta and gamma frequency bands that exhibited significant differences between the IA and HC groups, with the IA group manifesting an elevated level of phase synchronization. These findings align with emerging evidence that disruptions in neural connectivity and neurotransmitter systems in IA affect both the inhibitory and reward pathways (Chen, Dong, & Li, 2023), providing valuable insight into the neural mechanisms driving addictive behaviors.

## Neural mechanisms of IA

In the frontal area, notable connections were observed in the delta band, and these results are consistent with previous neurophysiological studies that examined IA (Y.J. Kim et al., 2017; J. Lee et al., 2014). Delta oscillations in the frontal regions are thought to reflect underlying neural mechanisms involved in signal detection and the maintenance of attentional focus, often activated during cognitively demanding or self-regulatory processes (Başar et al., 2001; Knyazev, 2007). Increased frontal delta activity has been observed in conditions that require enhanced internal cognitive processing and a reduced response to external distractions. This function is crucial in executive processes and impulse control, as it may help filter out irrelevant sensory information, allowing for greater focus on goal-directed behavior (Harmony, 2013; Knyazev, 2007). Elevated delta activity during rest among individuals with IA may signal the impaired initiation of their inhibitory control mechanism. This also aligns with a recent electrophysiological study which found that IA individuals showed stronger neural responses to irrelevant information in a Stroop task than the normal control (Lin et al., 2023).

The gamma band also exhibited numerous connections across the entire scalp, notably concentrated in the occipital region. Gamma oscillations are critical for high-level cognitive functions, including attention, memory integration, and impulse control, which are often compromised in IA. The abnormal gamma coherence observed in IA may signify an imbalance in dopaminergic systems related to reward processing and impulsive behaviors (Buzsáki & Wang, 2012). Specifically, disruptions in dopamine neurotransmission, which have been associated with heightened gamma band activity, could impair the neural circuits involved in self-control and impulse regulation (Yordanova et al., 2002). The dopamine system plays a key role in reward anticipation and reinforcement, and an imbalance in this system may reinforce maladaptive behaviors and reduce the brain’s capacity to inhibit addictive actions, thereby contributing to the compulsive tendencies characteristic of IA. Furthermore, Crick and Koch (1990) proposed that sustained visual input, such as that experienced by individuals with IA, could lead to structural reorganization in visual processing areas and contribute to heightened gamma activity, thus increasing the brain’s predisposition toward sensory sensitivity and potentially reinforcing habitual internet use.

Overall, the findings of aberrant neural dynamics in both delta and gamma bands observed in this study may reflect dysfunctions in inhibitory control and impulse regulation networks. Heightened delta and gamma coherence patterns suggest a maladaptive neural connectivity profile that likely contributes to the cognitive and behavioral challenges seen in IA. Excessive visual stimulation due to prolonged computer and/or internet use may potentially reshape neural network structures in resting states.

## Contributions of the classification methods in identifying IA

In the realm of the classification task, the SVM method consistently outperformed the other two classification methods (RF and *k*NN), reaching a classification rate of 86% when utilizing WPLI values as features. Even though the *t*-test indicated fewer connections in the theta, alpha, and beta bands, it was evident that incorporating features from these bands remained beneficial for the classification process. This finding highlights that these bands contain valuable information that can enhance the classification task.

To further validate our findings, we conducted additional classification experiments using features derived from individual frequency bands (theta, alpha, beta, gamma, and delta), focusing on the significant connections identified through statistical testing. The results, presented in Tables 4 and 5, showed that while features from individual bands contributed to classification performance, their accuracy was consistently lower compared to the combined feature set, as shown in Tables 2 and 3. Specifically, the combined feature set, incorporating significant connections from all frequency bands, achieved higher classification accuracy than any single band. This improvement was likely due to the complementary nature of information across frequency bands, where each band captured distinct neural dynamics. Integrating these features allowed the model to leverage the strengths of each band, consistent with prior research demonstrating the benefits of feature combination in enhancing predictive performance (Hou et al., 2011). Additionally, combining features reduced the risk of overfitting to band-specific noise, improving the model’s robustness and generalization. These findings underscore the advantage of integrating features across multiple frequency bands.

**Table 4.** Classification results for each frequency band using PLI values as the feature set

SVM classifier					
Band	Delta	Theta	Alpha	Beta	Gamma
Accuracy	0.67 ± 0.05	0.65 ± 0.09	0.72 ± 0.14	0.68 ± 0.09	0.70 ± 0.08
Sensitivity	0.52 ± 0.16	0.51 ± 0.21	0.72 ± 0.16	0.59 ± 0.14	0.62 ± 0.19
Specificity	0.80 ± 0.14	0.78 ± 0.20	0.72 ± 0.17	0.76 ± 0.14	0.76 ± 0.05
TN	8.00 ± 1.41	7.80 ± 2.04	7.20 ± 1.72	7.60 ± 1.36	7.60 ± 0.49
FP	2.00 ± 1.41	2.20 ± 2.04	2.80 ± 1.72	2.40 ± 1.36	2.40 ± 0.49
FN	4.00 ± 1.26	4.20 ± 2.04	2.40 ± 1.36	3.40 ± 1.02	3.20 ± 1.60
TP	4.40 ± 1.36	4.20 ± 1.60	6.00 ± 1.26	5.00 ± 1.41	5.20 ± 1.60
kNN classifier					
Accuracy	0.67 ± 0.09	0.71 ± 0.09	0.74 ± 0.07	0.67 ± 0.08	0.68 ± 0.11
Sensitivity	0.53 ± 0.29	0.62 ± 0.19	0.72 ± 0.11	0.57 ± 0.18	0.60 ± 0.20
Specificity	0.80 ± 0.13	0.78 ± 0.15	0.76 ± 0.12	0.76 ± 0.05	0.76 ± 0.08
TN	8.00 ± 1.26	7.80 ± 1.47	7.60 ± 1.20	7.60 ± 0.49	7.60 ± 0.80
FP	2.00 ± 1.26	2.20 ± 1.47	2.40 ± 1.20	2.40 ± 0.49	2.40 ± 0.80
FN	4.00 ± 2.45	3.20 ± 1.60	2.40 ± 1.02	3.60 ± 1.36	3.40 ± 1.74
TP	4.40 ± 2.33	5.20 ± 1.47	6.00 ± 0.89	4.80 ± 1.60	5.00 ± 1.67
RF classifier					
Accuracy	0.61 ± 0.06	0.61 ± 0.15	0.73 ± 0.11	0.60 ± 0.07	0.69 ± 0.05
Sensitivity	0.38 ± 0.13	0.50 ± 0.19	0.71 ± 0.10	0.57 ± 0.11	0.53 ± 0.14
Specificity	0.80 ± 0.00	0.70 ± 0.13	0.74 ± 0.14	0.62 ± 0.15	0.82 ± 0.12
TN	8.00 ± 0.00	7.00 ± 1.26	7.40 ± 1.36	6.20 ± 1.47	8.20 ± 1.17
FP	2.00 ± 0.00	3.00 ± 1.26	2.60 ± 1.36	3.80 ± 1.47	1.80 ± 1.17
FN	5.20 ± 1.17	4.20 ± 1.72	2.40 ± 0.80	3.60 ± 0.80	4.00 ± 1.41
TP	3.20 ± 1.17	4.20 ± 1.60	6.00 ± 0.89	4.80 ± 1.17	4.40 ± 1.02

**Table 5.** Classification results for each frequency band using WPLI values as the feature set

SVM classifier					
Band	Delta	Theta	Alpha	Beta	Gamma
Accuracy	0.73 ± 0.06	0.63 ± 0.10	0.63 ± 0.08	0.65 ± 0.10	0.76 ± 0.08
Sensitivity	0.76 ± 0.12	0.71 ± 0.11	0.52 ± 0.16	0.62 ± 0.07	0.75 ± 0.18
Specificity	0.70 ± 0.11	0.56 ± 0.12	0.72 ± 0.12	0.68 ± 0.22	0.76 ± 0.19
TN	7.00 ± 1.10	5.60 ± 1.20	7.20 ± 1.17	6.80 ± 2.23	7.60 ± 1.85
FP	3.00 ± 1.10	4.40 ± 1.20	2.80 ± 1.17	3.20 ± 2.23	2.40 ± 1.85
FN	2.00 ± 1.10	2.40 ± 0.80	4.00 ± 1.41	3.20 ± 0.75	2.00 ± 1.41
TP	6.40 ± 1.02	6.00 ± 1.10	4.40 ± 1.50	5.20 ± 0.40	6.40 ± 1.85
kNN classifier					
Accuracy	0.70 ± 0.12	0.57 ± 0.06	0.65 ± 0.10	0.60 ± 0.08	0.68 ± 0.11
Sensitivity	0.75 ± 0.18	0.57 ± 0.10	0.52 ± 0.20	0.50 ± 0.09	0.47 ± 0.24
Specificity	0.66 ± 0.12	0.58 ± 0.07	0.76 ± 0.05	0.68 ± 0.17	0.86 ± 0.10
TN	6.60 ± 1.20	5.80 ± 0.75	7.60 ± 0.49	6.80 ± 1.72	8.60 ± 1.02
FP	3.40 ± 1.20	4.20 ± 0.75	2.40 ± 0.49	3.20 ± 1.72	1.40 ± 1.02
FN	2.20 ± 1.60	3.60 ± 0.80	4.00 ± 1.67	4.20 ± 0.75	4.40 ± 1.85
TP	6.20 ± 1.17	4.80 ± 0.98	4.40 ± 1.74	4.20 ± 0.75	4.00 ± 2.10
RF classifier					
Accuracy	0.72 ± 0.09	0.62 ± 0.10	0.64 ± 0.03	0.76 ± 0.09	0.68 ± 0.04
Sensitivity	0.65 ± 0.11	0.54 ± 0.06	0.59 ± 0.15	0.77 ± 0.12	0.54 ± 0.10
Specificity	0.78 ± 0.15	0.68 ± 0.15	0.68 ± 0.15	0.76 ± 0.19	0.80 ± 0.06
TN	7.80 ± 1.47	6.80 ± 1.47	6.80 ± 1.47	0.76 ± 0.19	8.00 ± 0.63
FP	2.20 ± 1.47	3.20 ± 1.47	3.20 ± 1.47	2.40 ± 1.85	2.00 ± 0.63
FN	3.00 ± 1.10	3.80 ± 0.40	3.40 ± 1.20	2.00 ± 1.10	3.80 ± 0.75
TP	5.40 ± 0.80	4.60 ± 0.80	5.00 ± 1.41	6.40 ± 0.80	4.60 ± 1.02

To provide context for our findings, we compared our results with recent studies that employed machine learning approaches to classify IA or related conditions, as summarized in Table 6. For instance, Sun et al. (2022) used convolutional neural networks to classify IA based on EEG spectral power data, achieving accuracies of 87.59% with full spectral power and 81.1% with raw EEG data. Wang et al. (2021) utilized fMRI-based functional connectivity density (FCD) features, achieving 82.5% accuracy with the SVM classifier. Similarly, Hsieh et al., (2019) developed an ensemble classifier combined with case-based reasoning (CBR) to categorize IA severity levels, exploring Internet usage patterns in temporary internet files (TIF) from personal computers and achieving an accuracy of 89.9%. While these studies offered valuable insights, they primarily focused on spectral power, fMRI connectivity density, or behavioral metrics, without emphasizing phase synchronization and its neurophysiological significance.

**Table 6.** Comparison of classification methods and feature sets in various IA studies

Reference	Participants	Data	Models	Features	Accuracy
Our study	42 IA individuals, 50 Healthy controls	RS-EEG	SVM, kNN, RF	PLI, wPLI	86%
Sun et al. (2022)	24 IA individuals, 25 Healthy controls	RS-EEG	CNN	Raw data, spectrum power	87.59%
Wang et al. (2021)	27 IA individuals, 30 Healthy controls	RS-fMRI	SVM	FCD	82.5%
Hsieh, Shih, Shih, and Lin (2019)	114 mild level, 91 moderate level, 12 severe level	Temporary Internet Files (TIF)	EMBAR	Dataset clustered by SOM	Mild: 86.3% Moderate: 84.9% Severe: 98.6%

Compared to previous studies, our research achieved a classification accuracy of 0.86 using EEG functional connectivity features and machine learning techniques, offering several notable contributions. We employed PLI and WPLI for robust phase synchronization analysis, minimizing volume conduction effects and setting our work apart from studies focused on spectral power or fMRI connectivity density. Our approach leverages cost-effective, noninvasive EEG data combined with machine learning, making it practical for real-time and large-scale IA assessments. Additionally, our lower-complexity machine learning model enhances resource efficiency and applicability. By examining functional connectivity across multiple frequency bands, particularly delta and gamma, we uncover richer insights into IA-related neural dynamics. The use of SVM with WPLI features further highlights the effectiveness of combining advanced phase synchronization metrics with machine learning. Overall, our study provides a practical, efficient, and insightful framework for improving IA classification and understanding its neural basis.

To date, few studies have explored phase information in the investigation of IA. As mentioned, we utilized the PLI and WPLI as metrics to assess the degree of phase synchronization in this study. Phase synchronization has previously been acknowledged as pivotal in the investigation of conditions such as dyslexia (Fraga González et al., 2018) and Alzheimer’s disease (Knyazeva et al., 2010). Through rigorous statistical analysis and the application of machine learning techniques, we demonstrated the pivotal role of phase synchronization in individuals with IA. The PLI and WPLI values obtained in our study indicate that the phase information of delta and gamma bands may serve as potential biomarkers for identifying IA.

Notably, phase synchronization biomarkers have also gained recognition in diverse clinical applications. For instance, they have been used to distinguish responders to vagus nerve stimulation in pediatric epilepsy using SVM classifiers (Ma et al., 2022) and identify unipolar and bipolar depression through feature fusion of PLV, PLI, and WPLI (Duan et al., 2021). Additionally, WPLI has been utilized as an EEG functional connectivity feature in mild stroke patients, achieving high accuracy with SVM-RFE (support vector machine recursive feature elimination) (Xu et al., 2023). These advancements highlight the potential of combining phase synchronization metrics with machine learning, paving the way for future research to integrate these approaches in IA studies and further expand on the promising findings presented here.

Although IA has been extensively explored by numerous scholars, it is crucial to note that not all facets of Internet use have detrimental effects. Chou and Hsiao (2000) conducted a survey among Taiwanese students, revealing that the majority of the participants perceived the positive impacts of Internet use on their peer relationships. Social media, in particular, served as an additional means of communication, facilitating the sharing of experiences and collaborative online gaming, which fostered common interests and topics among peers. The Internet offers users a temporary escape from real-life stressors, providing a sense of enjoyment in the virtual realm. Moreover, the platform allows for anonymity, enabling individuals to freely express their thoughts without inhibition. These positive dimensions of the Internet contribute to its multifaceted influence on individuals’ lives. Therefore, the question of whether IA should be classified as a disease remains a topic worthy of discussion. Understanding the underlying factors contributing to IA and promoting a healthy attitude toward Internet use among adolescents could be pivotal in preventing and addressing this issue.

## Limitation and conclusion

Even though EEG provides the advantage of high temporal resolution, its spatial resolution is constrained, posing challenges in pinpointing the exact brain regions involved in IA. To address this limitation, future research could integrate additional structural or functional imaging techniques, such as functional magnetic resonance imaging (fMRI) or magnetoencephalography (MEG). Combining these modalities may enable a more comprehensive understanding of the neural mechanisms underlying IA, ultimately enhancing clinical diagnosis and treatment strategies.

Furthermore, the criteria for defining IA must be taken into consideration. Although the CIAS score of 64 is empirically validated as a diagnostic threshold for IA and demonstrates high diagnostic accuracy, specificity, and reliability, its limitations should be acknowledged. This threshold may not fully capture the heterogeneity of IA across diverse populations and contexts, as it was primarily validated in adolescent and college student populations (Ko et al., 2009). Consequently, the generalizability of this threshold to other demographic groups, such as older adults or individuals from different cultural backgrounds, remains uncertain, underscoring the need for further validation to ensure applicability across diverse subpopulations. Moreover, IA is a complex condition shaped by psychological, behavioral, and environmental factors, and relying solely on this threshold may oversimplify the diagnostic process, overlooking individuals exhibiting IA-related behaviors below the threshold. Incorporating complementary assessments, such as clinician-administered interviews or additional psychometric tools, could provide a more nuanced evaluation. Additionally, the current threshold does not account for variations in symptom severity or their effects on neural and behavioral outcomes. Future research should explore dynamic and flexible diagnostic criteria that integrate symptom severity and contextual factors to improve diagnostic accuracy and comprehensiveness.

Another important consideration is the role of Internet use patterns—such as duration, frequency, device type, and content—in understanding IA and its relationship with EEG changes. Hsieh et al. (2019) categorized IA severity using temporary Internet files, achieving 89.9% accuracy. These files, which store cached data, reflect key usage patterns tied to IA. In contrast, our study examined EEG functional connectivity using PLI and WPLI, achieving 86% accuracy in distinguishing IA from non-IA subjects. While Internet use patterns were not analyzed here, their potential link to EEG changes and used for distinguishing between IA and non-IA subjects remains an interesting topic for future research. Further studies could also help control confounding variables more effectively.

Finally, we acknowledge that the observed EEG differences could be influenced not only by Internet use itself but also by secondary effects such as sleep deprivation, reduced physical activity, or changes in social interactions. While beyond the scope of this study, disentangling these direct and indirect effects warrants further investigation. Addressing these limitations through advanced imaging modalities, refined diagnostic frameworks, and broader assessments of lifestyle factors could strengthen future research and contribute to more effective prevention, diagnosis, and treatment strategies for this increasingly prevalent condition.
